# The role of positive spirituality in preventing child maltreatment and promoting resilience: a perspective on policy and practice

**DOI:** 10.3389/fpubh.2025.1656760

**Published:** 2025-09-16

**Authors:** Sabine Van Tuyll Van Serooskerken Rakotomalala, Katy Anis, Susan Hillis, Sydney Tucker, Xiaoan Li, Marijn Stok

**Affiliations:** ^1^Department of Interdisciplinary Social Science, Utrecht University, Utrecht, Netherlands; ^2^Fetzer Institute, Kalamazoo, MI, United States; ^3^Department of Social Policy and Intervention, Global Reference Group for Children Affected by Crisis, University of Oxford, Oxford, United Kingdom; ^4^Department of Mathematics, Imperial College London, University of Oxford, Oxford, United Kingdom; ^5^Department of Social Policy and Intervention Evaluation, University of Oxford, Oxford, United Kingdom; ^6^University of California Berkeley, Berkeley, CA, United States

**Keywords:** positive spirituality, violence prevention, resilience, faith-based approaches, child maltreament

## Abstract

Child maltreatment remains a pervasive global issue with profound impacts on health, development, and societal well-being. While evidence-based strategies for violence prevention have expanded, the role of spirituality as a protective and rehabilitative factor remains underexplored in mainstream policy and practice. This perspective article examines how nurturing spiritual values across the life course—such as empathy, emotional regulation, and ethical decision-making—can contribute to violence prevention and resilience building. Drawing on recent empirical findings and global policy examples, the paper argues for the integration of positive spirituality into child protection and faith-based initiatives across home, school, and community settings. The article outlines actionable strategies for practitioners and policymakers, highlights culturally responsive measurement tools, and calls for strengthened collaboration between child protection and faith-based sectors. By advancing a holistic and values-based approach to caregiving, this perspective contributes to ongoing efforts to disrupt intergenerational cycles of violence and promote children’s well-being in low-, middle-, and high-income countries worldwide.

## 1 Introduction

Child maltreatment is a major global public health issue, affecting about 1 in 2 children aged 2–17 each year (1, 2). It includes physical, sexual, and emotional abuse, as well as neglect, and can be perpetrated by parents, peers, teachers, intimate partners, or others. These harms occur in various settings, including the home, schools, online spaces, communities, and institutions. Research is complicated by underreporting and varying definitions across countries (3). Yet, global estimates remain alarming: 12.7% of children experience sexual abuse, 22.6% physical abuse, 36.3% emotional abuse, 16.3% physical neglect, and 18.4% emotional neglect (4). Girls are more likely to experience sexual violence—around 20%, compared to 10% of boys—especially in school settings, while boys are more frequently subjected to physical violence such as corporal punishment (5). Child maltreatment is linked to a range of direct and indirect adverse outcomes, such as injury and death (6, 7) as well as unintended pregnancy, mental illness, substance abuse and premature mortality (8–11). Economically, its burden is substantial, accounting for up to 4.3% of annual global GDP (12). Violence often extends across generations; however, intergenerational transmission is not inevitable. Not all maltreated individuals go on to perpetrate abuse, as outcomes are mediated by broader ecological factors (13).

Over the past decade, initiatives to prevent violence against children have grown substantially, partly driven by the adoption of the Sustainable Development Goals (SDGs) Target 16.2 and the publication of the INSPIRE Framework, which outlines seven core strategies to prevent such violence (14). Using an acronym, the INSPIRE strategies are Implementation and enforcement of laws; Norms and values; Safe environments; Parent and caregiver support; Income and economic strengthening; Response and support services; and Education and life skills. Building on the INSPIRE framework and existing research on spirituality and health, this perspective article examines the potential of strengthening spirituality as a means of preventing child maltreatment and supporting the rehabilitation of survivors. In doing so, it defines spirituality as distinct from organized religion, recognizing it as a broader, more inclusive dimension of human experience. While religion refers to structured systems of beliefs and practices aimed at connecting with the transcendent (15), spirituality is broader and may exist outside formal religious affiliation. Some individuals identify as spiritual without belonging to a specific religion, although spirituality can also be expressed through various religious traditions, including Buddhism, Christianity, Hinduism, Islam, Judaism, and Indigenous belief systems (16). This article focuses on nurturing positive spiritual development in children—an approach that transcends religious boundaries and applies across cultures and belief systems (17). Specifically, it proposes avenues through which policy makers, parents, faith leaders, educators, and child protection professionals can integrate spirituality into caregiving relationships through informed policies and practices. The central thesis posits that fostering spiritual development cultivates key life skills—including, for example, empathy, emotional regulation, critical reflection, and conflict resolution—which are essential for reducing the likelihood of violence in caregiving contexts. When nurtured in and by parents and caregivers, these spiritual competencies can enhance stress management and promote nonviolent, nurturing environments. In turn, children exposed to these values may internalize them, contributing to a disruption in the cycle of intergenerational violence and fostering community norms anchored in solidarity and compassion. The target audience for this perspective article is policymakers and professionals working on the prevention of violence against children (i.e., social service, health, education, and justice professionals) as well as faith leaders. Elucidating the relationship between the cultivation of spiritual values in childhood and their role as a protective factor for both perpetrators and victims of violence may enable the expansion of evidence-based initiatives within homes, schools, and communities, as well as contribute to the advancement of research in this domain.

The paper proceeds by defining and reporting key measures of positive spirituality and resilience, reviewing the empirical evidence supporting spirituality as a protective and rehabilitative factor, outlining actionable strategies for policymakers and professionals, and concluding with a discussion on next steps for integrating faith-based and child protection sectors to advance child well-being.

## 2 Defining and measuring positive spirituality and resilience

**Figure 1:**
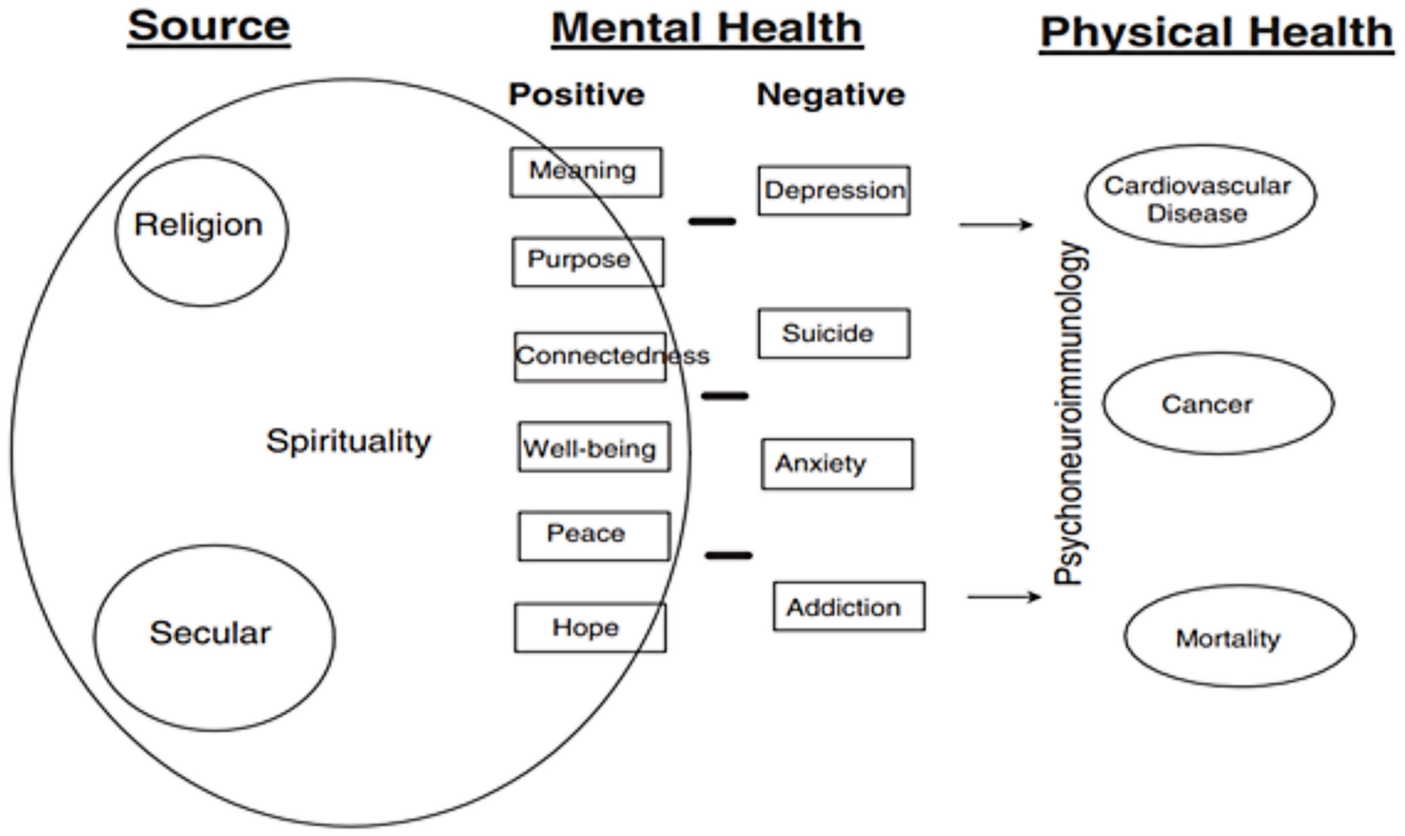
Definition of spirituality and its relationship to mental and physical health (22).

As of 2020, about 75.8% of the global population identifies with a religious or spiritual tradition; this estimate is based on data from the Pew Research Center, which includes over 2,700 censuses and surveys from 230 countries (18). Positive spirituality is broadly defined as the quest for meaning, purpose, and transcendence (19) and is increasingly recognized as an important internal resource that underpins psychological resilience (20, 21). Spiritual development is both globally relevant and culturally diverse, occurring both within and beyond the confines of organized religion (15, 16). As outlined by Koenig (22) in Figure 1, spirituality is increasingly linked to mental and physical health. However, its definition has broadened to include not only deeply religious individuals but also secular seekers of well-being. Modern tools for transmitting and measuring spirituality often incorporate traits such as optimism, gratitude, and purpose, which are also recognized as indicators of good mental health (22).

Before further defining it, it is important to note that spiritual development is neither linear nor universally positive. While it can foster values such as moral integrity, emotional well-being, and resilience, it can also, under certain conditions, engender intolerance, discrimination, or reinforce harmful norms and practices (23–27). Acknowledging these complexities and potential negative outcomes, the current discussion focuses on the positive contributions that child protection professionals and spiritual leaders can make in cultivating nonviolent, nurturing environments for children.

Drawing on the work of the Consortium on Nurturing Values and Spirituality in Early Childhood, along with established social–emotional learning frameworks (28) and recent literature reviews (29), five key domains of spiritual values and capacities have been identified below. When nurtured, these domains may support mental health, strengthen resilience, and help reduce violence. The categories are not rigid divisions; many of these values and capacities are interrelated. For example, a child, youth, or adult who has developed self-awareness and relationship skills may devise an alternative option to resolve a conflict rather than resorting to violence.

1. Self-awareness: This involves recognizing personal emotions and understanding the relationship between thoughts, feelings, and behaviors. Related concepts include mindfulness, self-discipline, purposefulness, and mastery of self.
2. Self-management: This encompasses adaptive behaviors such as relaxation techniques, positive self-talk, goal-setting, and intentional focus. Keywords associated with this domain include determination, detachment, self-regulation, and intentionality.
3. Social awareness: Refers to the ability to empathize, appreciate diversity, and engage with others from a place of compassion and curiosity. Keywords include wonder, gentleness, and imagination.
4. Responsible decision-making: Involves ethical problem-solving and discernment. Key values include respect, justice, peacefulness, and truthfulness.
5. Relationship skills: Reflect capacities for trust-building, altruism, and cooperation. Concepts include generosity, humility, community-mindedness, and gratitude.

Measuring spirituality, while inherently complex due to its subjective and culturally embedded nature, is increasingly feasible. Tools such as the Psycho-Spiritual Wellbeing Scale (P-SWBS) assess various dimensions, including connectedness, compassion, and transcendence (30). Spirituality-specific tools include the Daily Spiritual Experience Scale [DSES; (31)], the Spiritual Transcendence Index (32), and the Expressions of Spirituality Inventory (33). Contextually adapted instruments like the Wicozani Instrument, developed for Indigenous populations, further reflect the diversity in spiritual expression (34, 35). For adolescents, the Adolescent Spiritual Health Scale—validated across 12 countries—assesses spiritual connectedness and has demonstrated associations with positive mental health (36, 37). In clinical settings, the FICA Spiritual History Tool is also adapted for youth to evaluate spiritual identity, values, community involvement, and relevance to care (38).

## 3 Spirituality as a protective and rehabilitative factor

In a recently published article on the link between spirituality and health care, 166 articles were analyzed, revealing 24 commonly cited dimensions of spirituality, primarily related to connectedness and life meaning (39). Based on this, a framework was developed to quantify spirituality, supporting its relevance in healthcare research and practice (39). In complement to this research and framework, and building on the definitions of violence and spirituality, this section synthesizes empirical findings on the protective and rehabilitative roles of spirituality specifically in the context of child maltreatment. Drawing on a public health framework, the summary below is organized according to the three levels of prevention: primary (preventing onset), secondary (early intervention), and tertiary (rehabilitation) (2).

### 3.1 Primary prevention

Recent meta-analytic evidence reveals a consistent inverse association between spiritual or religious involvement and violent behaviors. Gonçalves et al. (40) found that over half (55.4%) of 101 outcomes across 269,910 individuals demonstrated that spirituality or religion significantly reduced the likelihood of physical and sexual aggression. Furthermore, a meta-analysis of 60 empirical studies found that religious beliefs and behaviors exert a deterrent effect on criminal behavior, including violence (41) and broad reviews of over 350 physical health and 850 mental health studies show that spiritual engagement is linked with lower levels of anxiety and depression—two key risk factors for violent behavior (42–44).

### 3.2 Secondary prevention

Spirituality also plays a crucial role in fostering resilience and promoting recovery following exposure to violence. Schwalm et al. (20) report a statistically significant positive correlation between spiritual engagement and resilience. Cultivating spiritual beliefs has been shown to moderate emotional responses to trauma, potentially interrupting intergenerational cycles of abuse (45, 46). Among survivors of sexual violence, those who experienced spiritual growth post-trauma demonstrated enhanced psychological recovery (47). Similarly, spiritual beliefs were found to enhance resilience and reduce trauma-related symptoms among survivors of violent trauma in an extensive community study (48).

### 3.3 Tertiary prevention

Engagement in religious communities and volunteerism has been associated with improved purpose and healing (49, 50). In humanitarian crises, spiritual support has been shown to alleviate stress and reduce the risk of violence among displaced populations (51–53), and in conflict-affected regions, spiritual resources have been linked to improved parenting capacity and reduced use of violent discipline (54–57).

## 4 Actionable strategies for practitioners and policymakers

Children’s development of spiritual values and capacities does not occur in isolation. Three foundational conditions are essential for nurturing these elements: a safe, nonviolent, and respectful environment; caring and positive relationships with parents, caregivers, and educators; and opportunities for children to practice and internalize their own spiritual development (58). Building on the growing evidence base that highlights spirituality as a valuable tool in violence prevention and response, this section presents practical, evidence-based strategies for integrating spiritual values and capacities at various levels, ranging from the policy level to family, education, child protection, and religious institutions.

Evidence suggests that, in the broader realm of child and family support, policies have consistently demonstrated an impact in promoting child well-being (59). While regional trends are challenging to identify, several countries provide illustrative examples of how spirituality can be integrated into child-focused frameworks. For instance, Malaysia’s Child Act mandates the inclusion of moral and spiritual considerations in child welfare policy (60), and Bhutan’s Gross National Happiness framework integrates spiritual values across multiple sectors (61, 77). Educational reforms in other nations have followed similar trends: Bolivia promotes Indigenous spiritual values in curricula (62), Kenya incorporates spiritual knowledge into school readiness assessments (63), and countries such as Malawi, Mauritius, and the Solomon Islands explicitly include spiritual development in early childhood education (64–66). Given the likelihood that policies will translate into legislation or codes of practice (67), these examples provide interesting case studies for aligning national policies with the spiritual dimensions of child well-being.

Shifting from policy to relationships, the role of the child’s immediate environment becomes central. When children are treated as spiritual beings—through safe, loving relationships that affirm their agency and sense of purpose—they are more likely to thrive (50). Thriving children are better equipped to make sound decisions, treat others with respect, and develop a strong sense of social responsibility (50). To effectively nurture spirituality in children, adults must also engage in their own spiritual growth (i.e., the metaphor of filling one’s own cup before pouring into others). In 2021, the World Health Organization commissioned two systematic reviews on parenting interventions, covering 435 randomized controlled trials across 65 countries (68). The findings showed that providing support to parents consistently leads to reductions in negative parenting behaviors, including maltreatment, and improvements in nurturing practices across all regions and types of interventions. The “Toolkit on Nurturing the Spiritual Development of Children in the Early Years” provides practical resources such as a “Learning Program for Adults” and an “Activities for Children” booklet to support this dual development (58).

Faith communities also play a powerful role. A scoping review of 172 sources on faith-based initiatives found that religious leaders are uniquely positioned to reinterpret harmful doctrines and instead promote beliefs that protect children and encourage responsive caregiving (69). The importance of this role was underscored during the COVID-19 pandemic, when the WHO advocated for the inclusion of faith actors in emergency response efforts due to their wide-reaching influence (70). While unstructured, everyday interactions are crucial for the development of spiritual values and capacities, structured strategies are also vital for preventing intentional violence. Evidence from socioemotional learning programmes—especially those implemented in schools using the Strengths Approach, a social justice-oriented framework (71)—demonstrates their potential to promote nonviolent behavior and reduce violence when caregivers and communities are actively involved (72–75). Effective programmes typically include five key components: (1) diverse learning tools such as videos, games, discussions, and visual aids; (2) repeated exposure through multiple lessons and ongoing reinforcement; (3) interactive peer activities like role-plays to encourage positive norms; (4) whole-school engagement, including leadership participation; and (5) active parental involvement through take-home materials rather than passive information sessions (29). By intentionally combining spiritual values, developmentally supportive relationships, and evidence-based programme structures, child protection practitioners can more effectively prevent violence and support recovery for affected children.

## 5 Discussion

The practical implications of this perspective, suggested below, are evidence-informed and multifaceted. On the research front, efforts should focus on developing validated and unified metrics that can meaningfully capture spiritual dimensions, distinct from indicators of religious attendance or mental health (e.g., the key domains outlined in Section Two: self-awareness, self-management, social awareness, responsible decision-making, and relationship skills). Greater investment is then needed in longitudinal and quasi-experimental research to explore how spiritual values and capacities are most effectively transmitted, and how they contribute to reducing violence over time (20, 40). At the policy level, global and regional initiatives should be strengthened to facilitate the exchange of knowledge and best practices for integrating spirituality into policy frameworks. At the family level, parenting and caregiver support programmes—shown to be effective across diverse regions (68)—should intentionally include the transmission of spiritual values and capacities, such as empathy and emotional regulation, which were identified in Section 2 as protective factors. Likewise, faith leaders—who research shows can shift harmful norms and promote nurturing beliefs (69)—should collaborate with educators and child protection workers through structured and culturally grounded initiatives such as camps, sermons, and school-based programmes that promote positive values and capabilities, contributing to the protection and well-being of children.

In line with the 2030 Agenda for Sustainable Development, which calls for global peace, justice, and strong institutions (76), it is essential to engage both governments and grassroots actors—parents, caregivers, educators, and faith leaders—in this shared mission. Fostering spiritual values and capacities is not only a means of preventing violence against children and upholding their rights but also a vital foundation for building societies rooted in peace, empathy, and shared responsibility.

## Data Availability

The original contributions presented in the study are included in the article/supplementary material, further inquiries can be directed to the corresponding author.
